# Supplementary material for: Charged biexciton polaritons sustaining strong nonlinearity in 2D semiconductor-based nanocavities
Source: Nat Commun. 2023 Aug 31;14:5310. doi: 10.1038/s41467-023-41079-y (PMC10471760; doi:10.1038/s41467-023-41079-y)
Supplement: Supplementary file 1 — Supplementary Information [file 41467_2023_41079_MOESM1_ESM.pdf]

Supplementary Information for

Charged biexciton polaritons sustaining strong nonlinearity in 2D semiconductor-based nanocavities

Supplementary Note 1: Sample preparation and characterization

Briefly, Monolayer WS_2 synthesized by chemical vapor deposition (CVD) methods was firstly transferred onto a precleaned fused silica substrate using wet transfer technique. Then periodic Ag nanodisk (ND, 100 μm × 100 μm) arrays were directly etched and deposited on top of WS_2 monolayer by means of electron-beam lithography and evaporation. Finally, a 200 nm thick polymethyl methacrylate (PMMA) layer was spin-coated onto the WS_2-Ag ND heterostructure to avoid sample degradation.

The array diameter is varied from 80 nm to 140 nm to acquire different plasmon resonances, with a height of 30 nm, while the period of the Ag array is fixed at 300 nm. At this array period, the non-local diffractive modes from Ag photonic crystal are sufficiently far away from the WS_2 exciton resonance, allowing a pure coupling between the exciton and localized plasmonic mode. The reflectance contrast spectrum, transient absorption spectrum and corresponding optical image of several typical samples are shown in Supplementary Fig. 1. For comparison, the reflectance contrast spectrum of the pure Ag ND is also provided. Zero detuning between the plasmon and the exciton resonance is roughly acquired at a ND diameter of 110 nm. Note that because of the moderate inhomogeneity of the Ag ND arrays, the plasmon resonance of pure Ag ND is not exactly consistent with that of WS_2-Ag ND heterostructure.

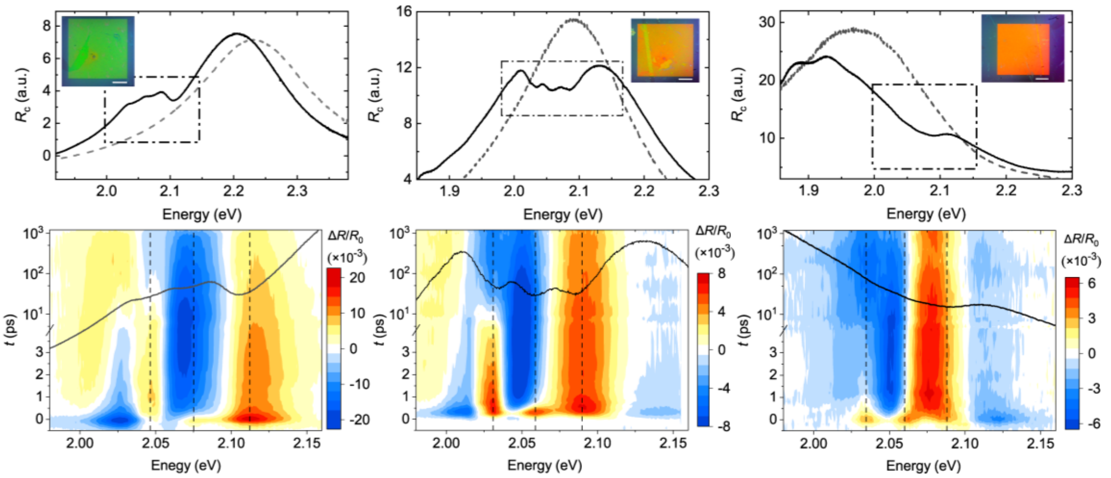

Supplementary Figure 1. Optical characterization of the hybrid sample with array diameter of 90 nm (left), 110 nm (middle) and 130 nm (right). Scare bar: 20 μm. The up panels show the reflectance contrast spectra of the WS_2-Ag ND (solid) and pure Ag ND (dash), with the sample optical images in the inset. The below panels show the corresponding transient absorption pseudo-color images of the WS_2-Ag ND under 2.195 eV pumping, overlapped by the zoomed-in reflectance contrast spectra indicated in up panel by dash dot rectangles.

Supplementary Note 2: PL spectrum, absorption spectrum and photo-absorption mechanism of the charged biexciton.

Upon being excited by 532 nm continuous laser at 4 K, the PL spectra of the monolayer WS_2 under two typical input power are shown in Supplementary Fig. 2a. Strong and broadband emission peak from

trap state is found below 2.0 eV. This peak is not found in the absorption spectrum (Supplementary Fig. 3) and transient absorption spectrum (Supplementary Fig. 1), but dominates the PL spectrum at low and medium input power, indicating the existence of a large number of trap states in the sample. These trap states may come from the doping of PMMA[1], the adsorption of gas molecules at the monolayer surface, or the sulfur atom vacancy during the preparation of WS_2. Regardless of the mechanism, these shallow trap states provide an abundant source of doped electrons for intrinsic WS_2 materials, similar to the Photogating effects in optoelectronics, which efficiently enhance the light absorption of charged exciton complexes such as trion (T, a three-particle state) and charged biexciton (XX^-, a five-particle state).

Four emission peaks are found above 2.0 eV, marked as X(~2.088eV), T(2.060eV), XX_D^- (2.039eV) and XX_B^- (2.028eV) (Supplementary Fig. 2b). The energy differences between the last three peaks and X are 28 meV, 49 meV and 60 meV, respectively. Compared with previous studies[2-5], these 4 peaks could be ascribed to the emission from exciton (X), trion(T), dark state charged biexciton (XX_D^-) and bright state charged biexciton (XX_B^-). The power-dependent PL intensity (Supplementary Fig. 2c) further confirms this ascription, which shows that X and T exhibit sublinear and linear increases with the input intensity, while XX_D^- and XX_B^- show superlinear behaviors with power-law exponents of 1.55 and 1.96, respectively. It is noted that although the neutral biexciton (XX, a four-particle state) also has a superlinear power-dependence, its binding energy is only ~20 meV[4,5], far from the value here. Therefore, we can safely rule out the contribution of XX to the PL emission (and also absorption) spectrum.

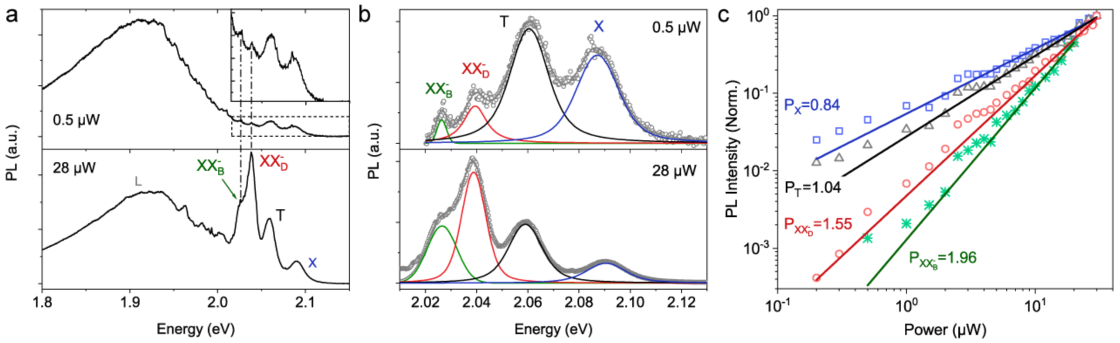

Supplementary Figure 2. Power-dependent PL spectrum of monolayer WS_2 at 4K excited by 532 CW laser. (a) Large energy-range PL spectra at two typical incident power. Inset of the up panel is the zoomed-in PL spectrum labeled by the dotted rectangle. (b) Corresponding high-energy region of the PL spectra and Voigt fitting curves after removing the trap state PL (labeled as L peak in (a)). (c) Power-dependent integrating PL of the 4 characteristic peaks marked in (b). Dots are experimental data, and lines are power-law fitting curves.

Compared with the diverse PL peaks, the absorption spectrum (Supplementary Fig. 3) of monolayer WS_2 under femtosecond supercontinuum white light excitation is much simpler. There are only three absorption peaks near A exciton resonance, which can be attributed to neutral exciton X (2.084eV), trion T (2.055eV) and charged biexciton XX^- (2.031eV). The absorption energy of T is ~29 meV lower than

X, coinciding well with the PL results. While the energy difference between XX^- and X is ~53 meV, a nearly intermediate value between that of XX_D^- (49meV) and XX_B^- (60meV), possibly arising from the hybrid absorption of the two charged biexciton states.

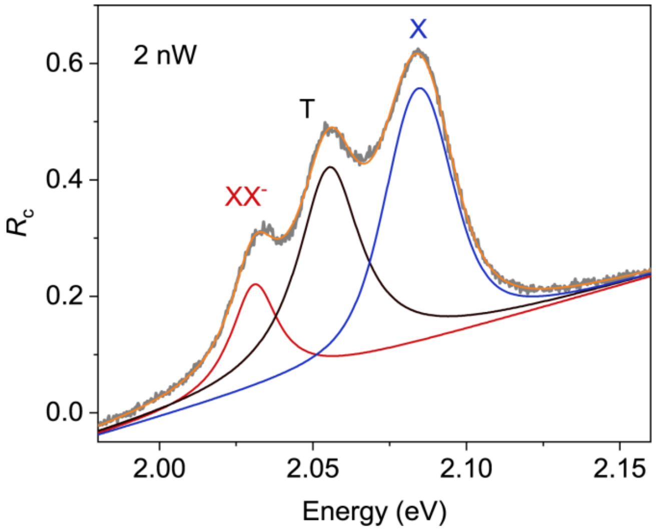

Supplementary Figure 3. Typical absorption spectrum of monolayer WS_2 at 4K. The experimental data (gray) is fitted (orange) by three Voigt peaks from X (blue), T (black) and XX^- (red) after subtraction of a linear background.

While PL emission from XX^- has been extensively reported in previous works, its absorption behavior is rarely observed. Very recently, Sarthak Das et al[3] reported a highly superlinear photocurrent generated in graphene (G)-WS_2 heterostructure, arising from strong absorption of two XX^- species in WS_2 (namely, XX_D^- and XX_B^-). Specifically, the G layer provides hot carrier doping to the adjacent WS_2 layer, and thus greatly enhances the oscillator strength of the charged biexciton. Generally, the formation process of XX^- can be understood as the successive absorption of two photons (with energy in resonance with the XX^- emission), and then the transition from the ground state to the XX^- excited state with the assistance of some extrinsic states. Since the energy of the XX^- is larger than the total energy of the two absorbed photons, with an energy gap of $\Delta = \hbar\omega_T - \hbar\omega_{XX^-} = 24\,\text{meV}$, energy conservation requires an energetically extrinsic state (e.g. trap state) to supplement this gap. In the case of G-WS_2 bilayer[3], the authors explain that such an energy gap might be provided by hot carrier doping (photothermionic emission) or interlayer absorption. Actually, apart from these two pathways, photogating effect from the large amount of shallow trap states at the sample surface may also provide excess electronic energy. As shown in Supplementary Fig. 4a, under light irradiation or thermal disturbance, energetic electrons are ionized from the shallow trap states[6,7], leading to the photodoping of the WS_2 and the enhancement of the optical absorption of the charged multi-particles. Recently, Shuai Fu et al[8] showed that the photogating effect from the trap state at the G/WS_2 interface can even lead to a long-life photoconductance in G layer.

One should note that the XX^- absorption cannot be simply regarded as a cascade absorption of $\hbar\omega_T$

and $\hbar\omega_{XX^-}$ photon (Supplementary Fig. 4b). This can be proved by the photocurrent excitation spectrum in the G-WS_2 heterostructure[3], in which the photocurrent peak is predominantly found at XX^- resonance. If the above cascade absorption works, the photocurrent peak should be governed by T resonance rather than XX^- resonance, since the generation of XX^- and thus the photocurrent primarily requires the absorption of a high-energy $\hbar\omega_T$ photon. In addition, XX^- absorption cannot be regarded as a two-photon absorption process (TPA, Supplementary Fig. 4c), for at least two reasons. Firstly, energy conservation dictates the absorption photon energy to be $\frac{1}{2}\left(\hbar\omega_{XX^-} + \hbar\omega_T\right) = 2.043$ eV for TPA, where no absorption peak is found. Secondly, the pump fluence used for TPA is much higher than that for single photon absorption, reaching at least a level of 80 μJ cm^{-2} at the heterostructure (see Supplementary Fig. 13) and thus 800 μJ cm^{-2} at the pure monolayer WS_2, considering the optical field amplification of the Ag plasmon. While here, for pure WS_2, only 1μJ cm^{-2} of pump fluence is used, so the TPA process can be safely excluded.

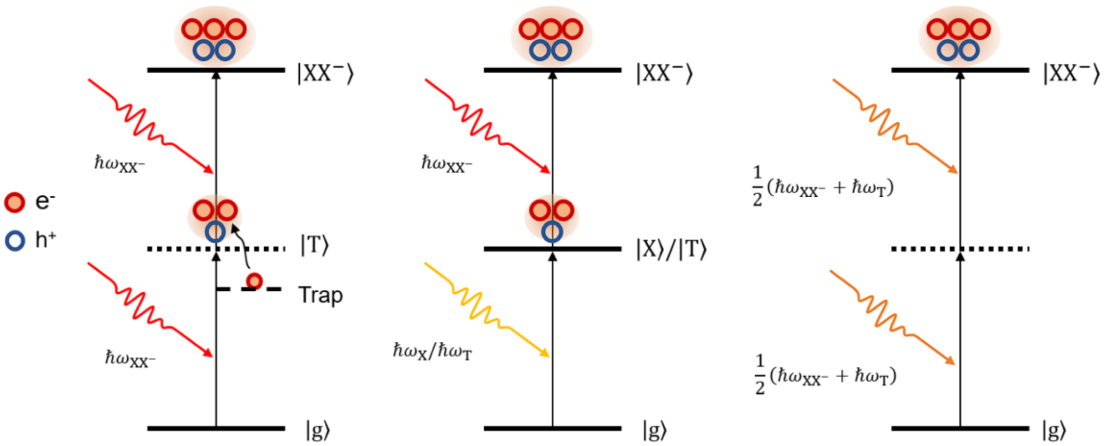

Supplementary Figure 4. Schematic diagram showing different XX^- absorption pathways. (a) Trap-assisted absorption. (b) Cascade absorption of a $\hbar\omega_X / \hbar\omega_T$ and a $\hbar\omega_{XX^-}$ photon. (c) Two-photon absorption.

Supplementary Note 3：Coupled oscillator model and fitting results

We use the classical coupled oscillator model to describe the interaction between different exciton species and plasmon. Since the plasmon line width (＞150 meV) is much larger than the energy gap between different excitons, X, T and XX^- will interact with the same plasmon mode. Considering that the coherent lifetime of polariton is only a few tens of femtoseconds, during which the nonlinear interaction between different exciton species should be much smaller than that within the same species, we thus assume that any interaction between different excitons can only be mediated by the plasmon. Moreover, unlike the dielectric microcavity polariton, whose photo-absorption mainly arises from the exciton component, the photo absorption of the nanocavity polariton is mainly attributed to the plasmon component, while the exciton absorption in pure WS_2 can be ignored. Therefore, the incident light field

first drives the oscillation of the plasmons and then the plasmons excite the excitons in WS_2, the rate equation of the whole coupled system is

$$\begin{aligned}
&\ddot{x}_{\text{pla}} + \gamma_{\text{pla}}\dot{x}_{\text{pla}} + \omega_{\text{pla}}^2 x_{\text{pla}} + \Omega_{\text{X}}\dot{x}_{\text{X}} + \Omega_{\text{T}}\dot{x}_{\text{T}} + \Omega_{\text{XX}^-}\dot{x}_{\text{XX}^-} = F_0 e^{-i\omega t} \\
&\ddot{x}_{\text{X}} + \gamma_{\text{X}}\dot{x}_{\text{X}} + \omega_{\text{X}}^2 x_{\text{X}} - \Omega_{\text{X}}\dot{x}_{\text{pla}} = 0 \\
&\ddot{x}_{\text{T}} + \gamma_{\text{T}}\dot{x}_{\text{T}} + \omega_{\text{T}}^2 x_{\text{T}} - \Omega_{\text{T}}\dot{x}_{\text{pla}} = 0 \\
&\ddot{x}_{\text{XX}^-} + \gamma_{\text{XX}^-}\dot{x}_{\text{XX}^-} + \omega_{\text{XX}^-}^2 x_{\text{XX}^-} - \Omega_{\text{XX}^-}\dot{x}_{\text{pla}} = 0
\end{aligned} \tag{S1}$$

where x, γ and ω are the displacement, linewidth and angular frequency, Ω_{X}, Ω_{T} and Ω_{XX^-} are the coupling strengths between plasmon-X, plasmon-T and plasmon-XX^-, respectively, $F_0 e^{-i\omega t}$ denotes the driving electric field.

By solving the above equations, the absorption/reflection coefficient of the polariton system can be written as

$$R_1(\omega) \propto \text{Im}\left(F_0 \dot{x}_{\text{pla}}\right) \propto \omega \,\text{Im}\left\{ \frac{S_{\text{X}} S_{\text{T}} S_{\text{XX}^-}}{S_{\text{pla}} S_{\text{X}} S_{\text{T}} S_{\text{XX}^-} - \omega^2 \Omega_{\text{X}}^2 S_{\text{T}} S_{\text{XX}^-} - \omega^2 \Omega_{\text{T}}^2 S_{\text{XX}^-} S_{\text{X}} - \omega^2 \Omega_{\text{XX}^-}^2 S_{\text{X}} S_{\text{T}}} \right\} \tag{S2}$$

where $S_j = \omega_j^2 - \omega^2 - i\gamma_j \omega$ (j = pla, X, T or XX^-).

Note that equation S2 describes a polariton system where all the plasmons in the reservoir have chances to couple with excitons. However, in our sample, the plasmons are primarily generated near the upper and lower edge rings of the Ag NDs (Supplementary Fig. 5c)[9]. Since the thickness of Ag NDs is as large as 30 nm, much larger than the interaction length between the plasmon and exciton (~10 nm)[10]. Therefore, only plasmons at the bottom edge ring can act as reservoirs to generate polaritons, and the reflection/absorption coefficient of their contribution can be well described by equation S2. While the plasmons at the upper edge ring are completely separated from the WS_2 layer, and their reflection/absorption coefficient can only be described by a Lorentz function, which is equivalent to the case of $\Omega_{\text{X}} = \Omega_{\text{T}} = \Omega_{\text{XX}^-} = 0$ in equation S2, i.e

$$R_2(\omega) \propto \omega \,\text{Im}\left\{ \frac{1}{S_{\text{pla}}} \right\} \tag{S3}$$

The total reflection/absorption coefficient at the WS_2-Ag NDs heterostructure can be expressed as

$$R(\omega) = R_{\text{pol}}(\omega) + R_{\text{pla}}(\omega) \propto kR_1(\omega) + (1-k)R_2(\omega) \tag{S4}$$

where $k \in (0 \sim 1)$ represents the polariton proportion, $R_{\text{pla}}(\omega)$ describes the contribution from the pure plasmon, and $R_{\text{pol}}(\omega)$ represents the contribution from the plasmon-exciton coupling system.

A typical reflectance contrast spectrum of the WS_2-Ag ND heterostructure is shown in Supplementary Fig. 5. We fit the R_c curve with and without considering the pure plasmon component using equation S4 and equation S2, respectively. The fitting curve without considering the plasmon component is far away from the experimental results, with the coupling strength of $\Omega_{\text{X}} = 55 \pm 4$ meV, $\Omega_{\text{T}} = 49 \pm 3$ meV, $\Omega_{\text{XX}^-} = 31 \pm 3$ meV, far less than that from the anticrossing behavior (88 meV, 80 meV and 70 meV for X, X- and XX^-, respectively. See Fig. 1 in the main text). In contrast, the fitting result can almost completely reproduce the experimental data after considering the plasmon component, and the coupling strength is $\Omega_{\text{X}} = 90 \pm 5$ meV, $\Omega_{\text{T}} = 73 \pm 8$ meV, $\Omega_{\text{XX}^-} = 41 \pm 8$ meV, which is comparable to that from anticrossing behavior. This undoubtedly confirms the necessity of considering the pure plasmon response in the reflection or absorption spectrum of the heterostructure.

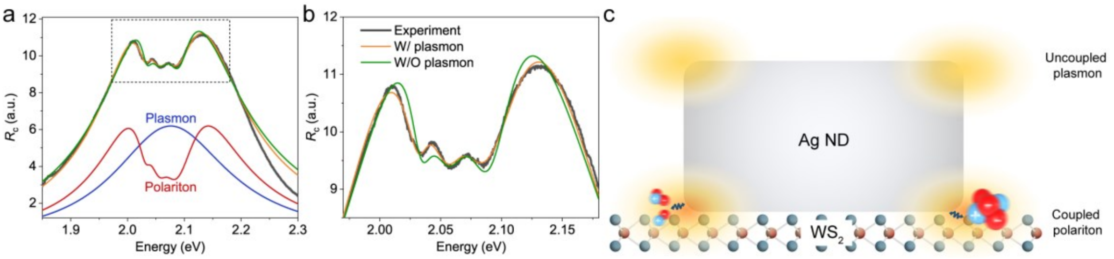

Supplementary Figure 5. (a) Representative reflectance contrast spectrum of the zero detuning WS_2-Ag ND heterostructure. Black line: experimental data, origin (green) line: Fitting result with (without) considering the contribution from pure plasmon component, blue line: pure plasmon component, red line: plasmon-exciton coupling component. (b) Zoomed in part of the dashed box in (a). (c) Schematic of the uncoupling and coupling states of the exciton-plasmon hybrid system. Only plasmon generated near the below ring of Ag ND can efficiently couple with the excitonic complex in WS_2.

We also fit the pump-power dependent reflectance contrast spectrum shown in Fig. 3a in the main text using the above model, and the results all present well consistent between the experimental and fitting curves, with $R^2 > 0.99$ for all power. All the fitting power-dependent parameters, apart from the ones presented in Fig. 3c-e, are shown in Supplementary Fig. 6 and Supplementary Fig. 7.

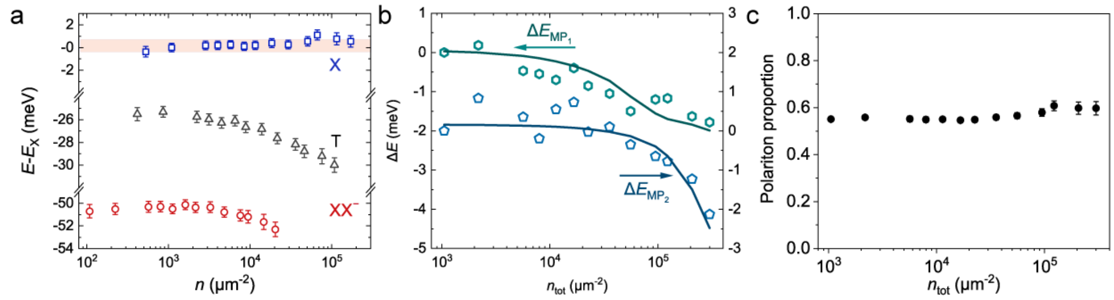

Supplementary Figure 6. Shifts of the (a) exciton species energy, (b) MP1 and MP2 energy and (c) polariton proportion as a function of the injecting carrier density, obtained from the fitting data in Fig. 3a in the main text.

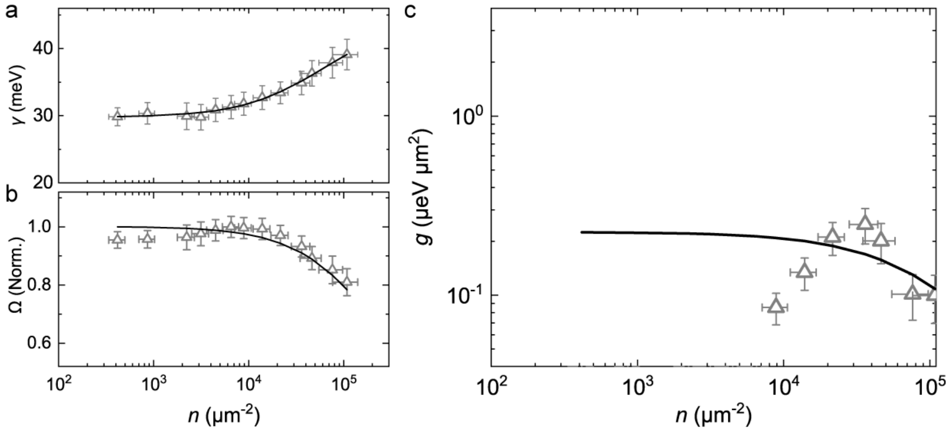

Supplementary Figure 7. Nonlinearity of the trion polaritons. Shifts of the (a) linewidth, (b) normalized coupling strength and (c) nonlinear coefficient of the trion polariton as a function of the injecting carrier density, obtained from the fitting data in Fig. 3a.

The polariton eigenfrequencies and Hopfield coefficients are also obtained by numerical solving the determinant of coefficient matrix of rate equation S1. For this analysis, we first measure the reflectance

contrast spectrum of the heterostructure with different detuning (Fig. 1c), and then extract the LP, MP1, MP2 and UP polariton energy. The plasmon energy $\hbar\omega_{\mathrm{pla}}$ can be obtained by using the relationship of $\omega_{\mathrm{LP}} + \omega_{\mathrm{MP1}} + \omega_{\mathrm{MP2}} + \omega_{\mathrm{UP}} = \omega_{\mathrm{pla}} + \omega_{\mathrm{X}} + \omega_{\mathrm{T}} + \omega_{\mathrm{XX^-}}$, with the exciton energy acquired from the reflection of monolayer WS_2[10]. Here, the shift of the excitonic resonance caused by sample inhomogeneity is neglected since it is much smaller than the energy change scale of polaritons in the dispersion curves (Supplementary Fig. 20). We then plot the dispersion curves (polariton energy versus plasmon energy), and fit them using the numerical solving polariton eigenfrequencies, as shown in Fig. 1d. The coupling strengths between the three excitons and plasmon are found to be $\Omega_{\mathrm{X}} = 88\ \mathrm{meV}$, $\Omega_{\mathrm{T}} = 80\ \mathrm{meV}$ and $\Omega_{\mathrm{XX^-}} = 70\ \mathrm{meV}$. The fitting Hopfield coefficients of the 4 polaritons versus the plasmon energy are shown in Supplementary Fig. 8, which all display intermixing behavior between the 3 excitons and plasmon, whose contributions depend on the plasmon−exciton detuning. We also performed the same experiment at room temperature, where only neutral exciton (X) couples with the plasmon, and the reflectance contrasts, the anticrossing curves and the Hopfield coefficients are presented in Supplementary Fig. 9. The fitting coupling strength is $\Omega_{\mathrm{X}} = 140\ \mathrm{meV}$, which is consistent with that at 4K, $\sqrt{\Omega_{\mathrm{X}}^2 + \Omega_{\mathrm{T}}^2 + \Omega_{\mathrm{XX^-}}^2} = 138\ \mathrm{meV}$. This indicates that all the oscillator strength of X exciton at room temperature is distributed to the X, T and XX^- states at 4 K.

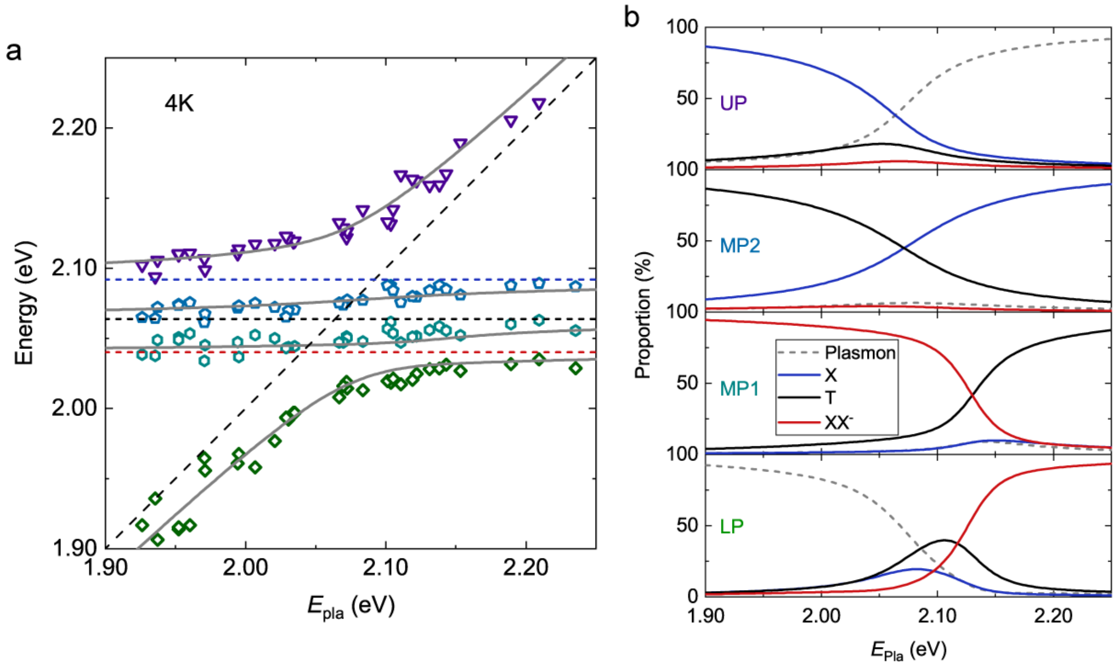

Supplementary Figure 8. Anticrossing behavior of the coupled system at 4K. (a) The 4 polariton (UP, MP2, MP1 and LP) energy as a function of plasmon resonance. Dots are extracted from the measured reflectance contrasts of the WS_2-Ag ND heterostructure with different detuning, solid lines are fitting curves using equation S1, and dash lines represent the 3 excitons and plasmon. (b) Hopfield coefficients for plasmon and the 3 exciton contributions to the UP, MP2, MP1 and LP states as a function of plasmon resonance.

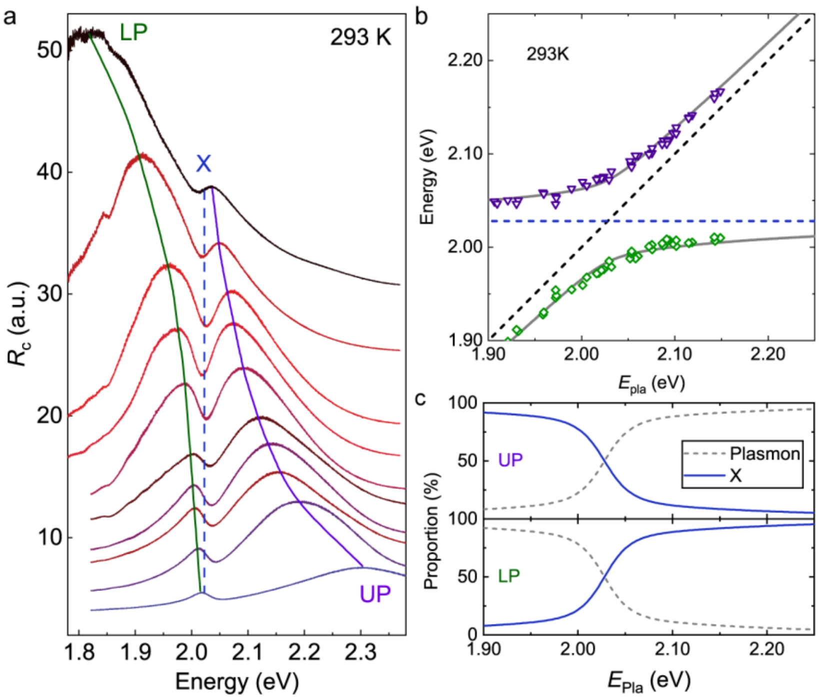

Supplementary Figure 9. Anticrossing behavior of the coupled system at 293K. (a) Reflectance contrast spectrum of the WS_2-Ag ND with different detuning. (b) UP and LP polariton energy as a function of plasmon resonance. Dots are extracted from (a), and solid lines are fitting curves using equation S1, and dash lines represent the X exciton and plasmon. (c) Hopfield coefficients for plasmon and the X exciton contributions to the UP and LP states as a function of plasmon resonance.

Supplementary Note 4：Calibration of the X, T and XX$^-$ polariton densities

4.1 Determination of absorption coefficient

The reflection (R) and absorption (A) spectra of the WS_2-Ag ND heterostructure are basically raised from the combination of the pure plasmon and the plasmon-exciton coupling systems. Here we only focus on the coupling component, namely $R_{\mathrm{pol}}(\omega)$ and $A_{\mathrm{pol}}(\omega)$. For the ultrathin layer on transparent quartz substrate investigated here, the reflectance contrast R_c is mainly determined by the imaginary part of the complex refractive index, that is, by the sample absorption. Thus, we can calculate $A_{\mathrm{pol}}(\omega)$ based on the reflectance contrast spectrum of the polariton component $R_{\text{c-pol}}(\omega)$, namely

$$A_{\mathrm{pol}}(\omega) = C \cdot R_{\text{c-pol}}(\omega) \tag{S5}$$

where C is the scale factor.

$R_{\text{c-pol}}(\omega)$ at different injected polariton densities are acquired by fitting the experimental R_c curves using equation S4, while the scale factor C can be obtained by simultaneously measuring the reflection R, transmission T_r and absorption $A = 1 - R - T_r$ spectra of the sample at room temperature, as shown in Supplementary Fig. 10. Note that since the reflectance of the quartz substrate is approximately constant, i.e. 3.5%, the reflectance R scales linearly with the reflectance contrast R_c, namely $R = 0.035(1+R_c)$.

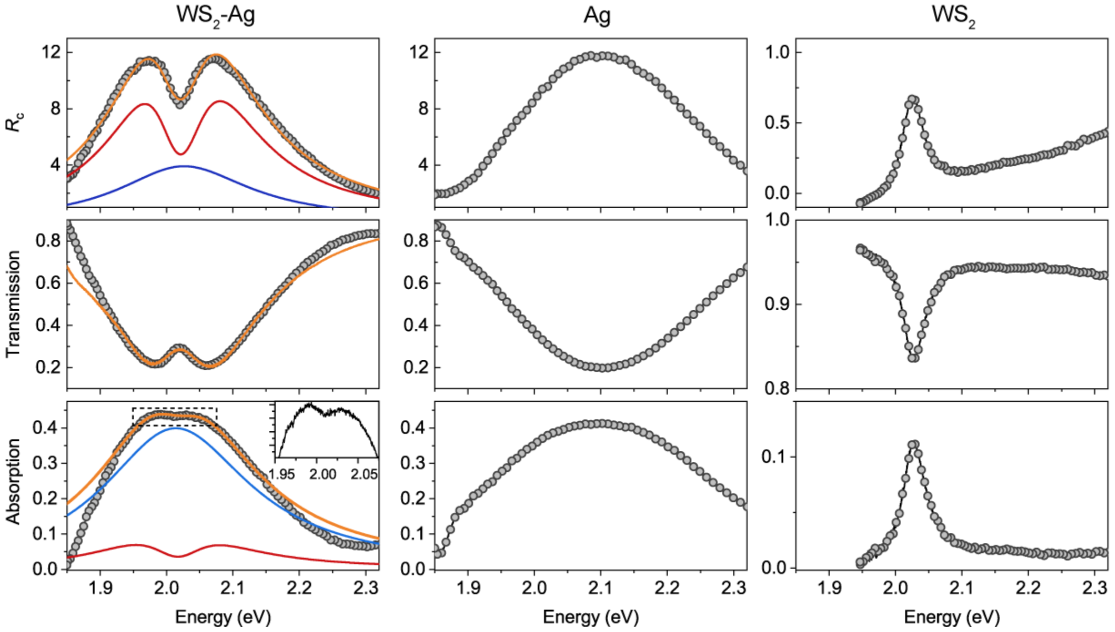

Supplementary Figure 10. Room temperature reflectance contrast, transmission and absorption spectra of the WS_2-Ag ND heterostructure, pure Ag ND and pure monolayer WS_2. The reflectance contrast and absorption spectra in WS_2-Ag ND region are fitted using equation S4. The blue, red and orange lines in the left panels represent the fitted spectra of pure plasmon, pure polariton and hybrid system.

It can be clearly seen that the shape of the reflectance contrast is dominated by the absorption features in both Ag ND and pure WS_2 regions. While these two spectra are different in WS_2-Ag ND, where $R_c(\omega)$ shows a much larger mode splitting than $A(\omega)$. This is caused by the difference of the polariton proportion (k) in these two spectra, as shown in the left panel of Supplementary Fig. 10. By fitting both $R_c(\omega)$ and $A(\omega)$ with equation S4, we can find that k = 70% in the reflection spectrum, while it drastically reduces to 16% in the absorption spectrum. These distinct polariton proportions are supposed to be originated from the stray scattering contribution in reflection spectra[11], which results in a more sizeable spectra dip in $R_c(\omega)$ than $A(\omega)$[2].

We extract the plasmon-exciton coupling components in $R_c(\omega)$ and $A(\omega)$, and compare them in Supplementary Fig. 11. Again, the shapes of these two curves are in excellent agreement, with a scale factor of $C \approx 0.8\%$. This means that a rise of unity in reflectance contrast of the coupling component corresponds to an increase of 0.8% in its absorption coefficient.

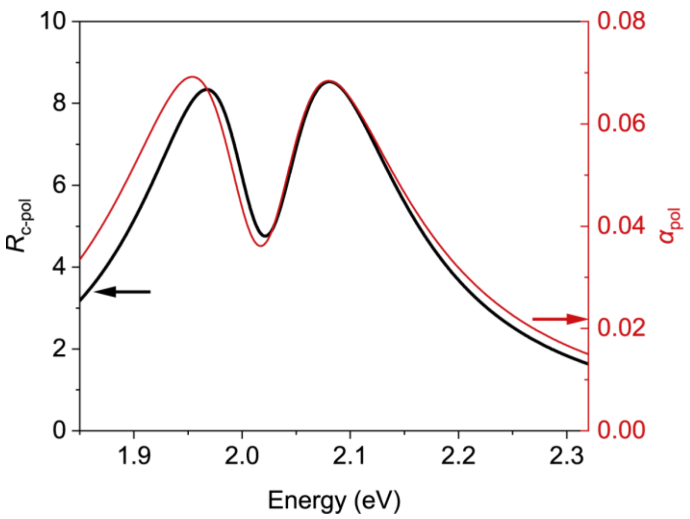

Supplementary Figure 11. Comparison of reflectance contrast and absorption spectra from the plasmon-exciton coupling component.

4.2 Calculation of the total polariton density n_{tot} and its uncertainty Δn_{tot} at different excitation intensities

The total polariton density can be calculated using

$$n_{\text{tot}} = \int_{E_{\min}}^{E_{\max}} \frac{LP(\omega)A_{\text{pol}}(\omega)}{fB_{\text{pol}} \cdot \hbar\omega} d\omega \quad \text{(S6)}$$

where $L \approx 70\%$ accounts for the transmission of the objective lens and the glass window of the cryogenic chamber, $P(\omega)$ is the intensity profile of the incident supercontinuum femtosecond laser, $f = 100$ kHz is the repetition rate, $\hbar\omega$ is the photon energy, B_{pol} is the polariton area exciting within a pump beam of $B_{\text{beam}} = 1.8\ \mu\text{m}^2$. In case of small plasmonic nanoparticles, B_{pol} is well approximated by the geometrical area of Ag NDs within the pump beam[12], i.e. $B_{\text{pol}} \approx \left(\frac{d_{\text{Ag}}}{p_{\text{Ag}}}\right)^2 B_{\text{beam}} \approx 0.13 B_{\text{beam}}$. The integral lower bound $E_{\min} = 2.016$ eV (615nm) is simply determined by the pump energy-dependent transient absorption spectrum, as shown in Supplementary Fig. 12 and Supplementary Fig. 13. More attention is thus paid to the integral upper bound. In previous study[13], we found that when the pump energy is high enough to generate the excited state excitons (such as 2S exciton), Coulomb screening effect from the hot carrier will greatly reduce the coupling strength of the polariton system. So here, $E_{\max} \approx E_{2s} = 2.234$ eV, corresponding to the energy of the lowest excited state exciton[14].

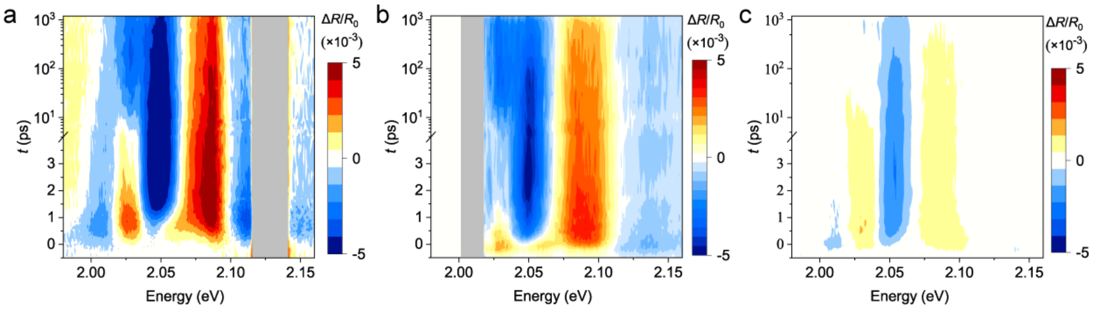

Supplementary Figure 12. Transient absorption 2D pseudo-color image of WS_2-Ag ND at different pump energy, with (a) 2.120 eV, resonant with UP, (b) 2.016 eV, resonant with LP, and (c) 1.984 eV, slightly below LP. Pump fluence is 10 μJ cm^{-2} in (a) and (b), and 80 μJ cm^{-2} in (c). The faint signal in (c) is caused by two-photon absorption confirmed by pump fluence-dependent transient absorption signal shown in Supplementary Fig. 13.

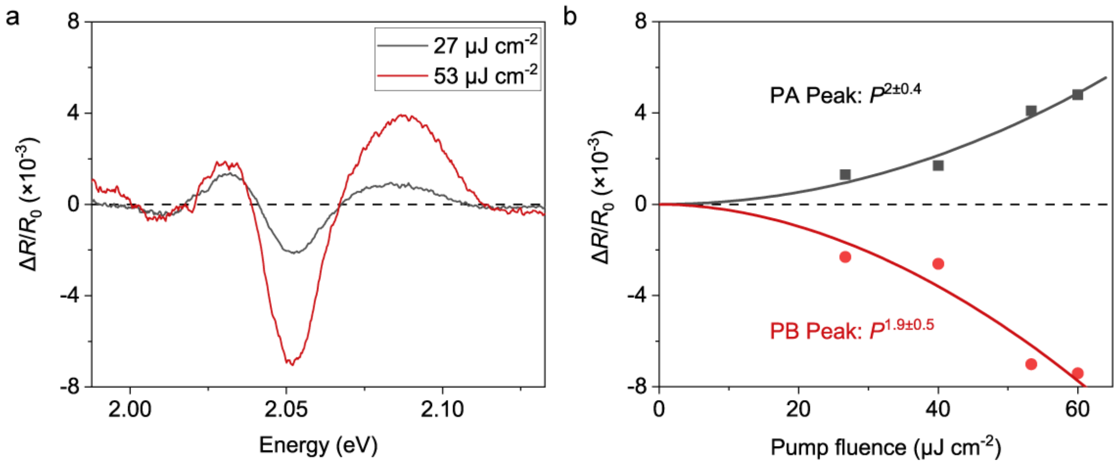

Supplementary Figure 13. Pump fluence-dependent transient absorption under 1.984 eV pumping. (a) Transient absorption spectrum at selected pump fluence. (b) Photo-induced absorption (PA) and bleaching (PB) intensities versus pump fluence. Dots are experimental data, and lines represent the power-law fits. The power exponent of 2 and 1.9 for the PA and PB peaks indicates a two-photon absorption for 1.984 eV pumping.

The uncertainty Δn_{tot} is deduced taking into account the errors in determining the incident power P, absorption coefficient A_{pol} and polariton area B_{pol}:

$$\left(\frac{\Delta n_{tot}}{n_{tot}}\right)^2 = \left(\frac{\Delta P}{P}\right)^2 + \left(\frac{\Delta A_{pol}}{A_{pol}}\right)^2 + \left(\frac{\Delta B_{pol}}{B_{pol}}\right)^2 \quad \text{(S7)}$$

All the fitting errors correspond to 95% confidence intervals.

4.3 X, T and XX^- polariton density

To quantitatively compare the nonlinearity of different exciton polaritons, we must disassemble the individual X, T and XX^- polariton density from the total density. Considering that the polariton

nonlinearity is essentially derived from the exciton component, we only need to consider the relative excitation efficiency of different excitonic complexes (η_X , η_T and η_{XX^-}) from plasmons in the heterostructure, which we assumed is consistent with that from photons in the pure WS_2 (that is, relative oscillator strength). As a result, the X, T and XX^- polariton density can be simply estimated by

$$\begin{aligned} n_X &= n_{tot} \cdot \eta_X \\ n_T &= n_{tot} \cdot \eta_T \\ n_{XX^-} &= \frac{1}{2} n_{tot} \cdot \eta_{XX^-} \end{aligned} \quad \text{(S9)}$$

where $\eta_{X/T/XX^-}$ is the relative oscillator strength of X/T/XX^- acquired from pure WS_2, with $\eta_X + \eta_T + \eta_{XX^-} = 1$, and the coefficient of 1/2 for n_{XX^-} accounts for the absorption of two photons to generate one XX^-. Note that $\eta_{X/T/XX^-}$ changes with injecting total exciton density due to different saturation nonlinearities of X, T and XX^- (Supplementary Fig. 14).

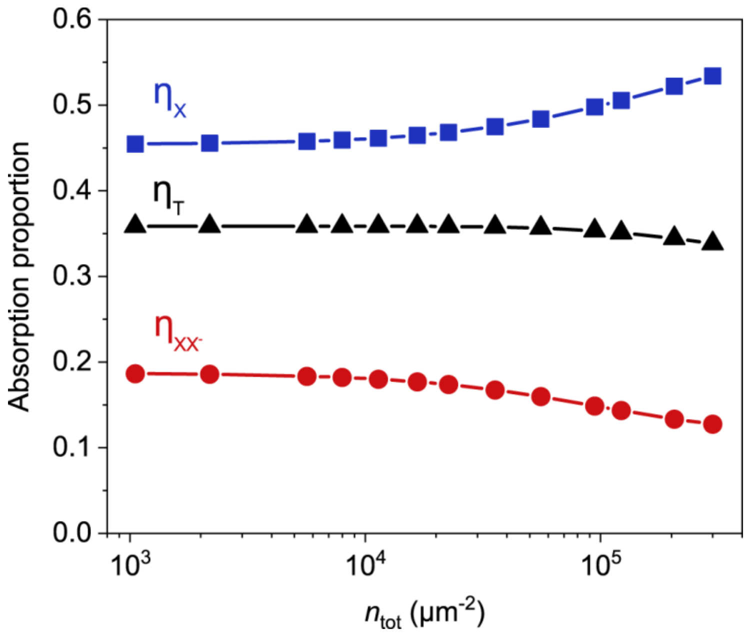

Supplementary Figure 14. Carrier density-dependent relative oscillator strength of X, T and XX^- extracting from fitting the reflectance contrast spectrum shown in Supplementary Fig. 15.

Supplementary Note 5：The nonlinear interaction of X, T and XX^- excitons in monolayer WS_2.

Since the nonlinearity of the polariton is essentially derived from the exciton component in WS_2, while the plasmon component in Ag ND can be ignored[11], the study of nonlinear interactions of different exciton species in pure WS_2 can provide a standard reference for that of polaritons in WS_2-Ag, and also offers a consistency check of our polariton density calibration.

The pure WS_2 absorption spectra under different injected total exciton densities (n_{tot}= 10^2-10^5 μm^{-2})

are shown in Supplementary Fig. 15a. Fitting the spectra with 3 Voigt peaks, the exciton density-dependent square root of absorption amplitude (corresponding to the coupling strength of the polariton in the heterostructure), linewidth and energy shift are acquired, as shown in Supplementary Fig. 15b-c. Obvious dephasing and saturation nonlinearities are found, which can be described using the phenomenological model in the main text, with $\alpha_X = (0.07 \pm 0.01)\ \mu eV\ \mu m^2$, $\alpha_T = (0.13 \pm 0.02)\ \mu eV\ \mu m^2$, $\alpha_{XX^-} = (0.5 \pm 0.1)\ \mu eV\ \mu m^2$, $n_{sX} = (8 \pm 1) \times 10^5\ \mu m^{-2}$, $n_{sT} = (2.3 \pm 0.2) \times 10^5\ \mu m^{-2}$ and $n_{sXX^-} = (2.0 \pm 0.2) \times 10^4\ \mu m^{-2}$. The broadening coefficients $\alpha_{X/T/XX^-}$ all agree well with previous reports[15], and the saturation intensity of the neutral exciton n_{sX} is in excellent agreement with $1/a_B^2 \sim 10^6\ \mu m^{-2}$. The saturation intensity ratio between X and XX^- is $n_{sX} / n_{sXX^-} = 40$, comparable to that of 56 in polariton system, indicating the same nonlinearity source of the two kinds of quasi-particles, and further verifying the high accuracy of the polariton density estimation.

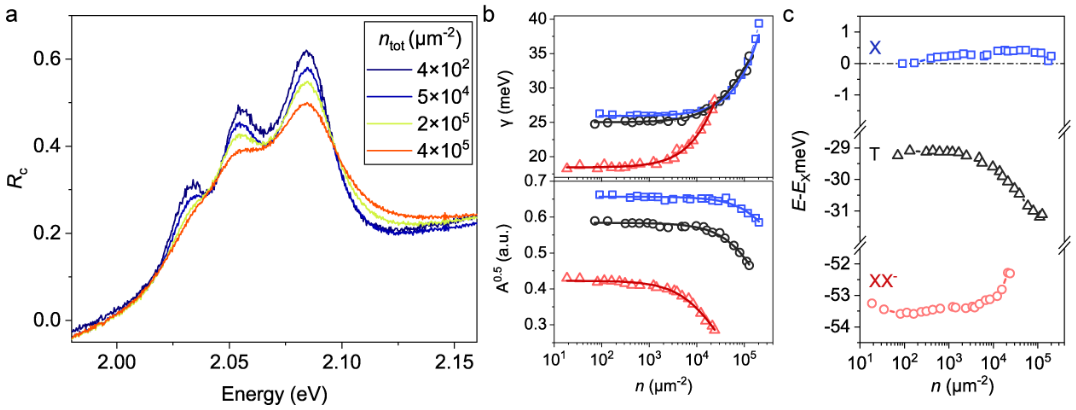

Supplementary Figure 15. Nonlinear interactions of X, T and XX^- in pure WS_2. (a) Exciton density-dependent reflectance contrast. (b) Exciton density-dependent linewidth (up panel) and square root of absorption amplitude (below panel). (c) Exciton density-dependent resonance energy. For comparison, the X energy at the lowest incident power is subtracted.

Supplementary Note 6: Estimation of the XX^- Bohr radius

6.1 Theoretical model

The Bohr radius or cross section of XX^- (a_{XX^-}) is roughly estimated using the kinetic theory relation as described by Yumeng You et al[16], where a charged biexciton is considered to be generated by the collision of an exciton and a trion, with the following rate equations:

$$\begin{aligned} \frac{dn_X}{dt} &= -\gamma_X n_X - \beta n_X n_T + \gamma_{XX^-} n_{XX^-} / (1+D) \\ \frac{dn_{XX^-}}{dt} &= -\gamma_{XX^-} n_{XX^-} + \beta n_X n_T \\ n_T &= D n_X \end{aligned} \tag{S10}$$

where β represents the XX^- formation rate from collisions of X and T, γ_X and γ_{XX^-} are the

recombination rates of X and XX^-, respectively, $D = 0.8$ is the density ratio of the T to X accounting for their different oscillator strengths but the same dynamics due to complete equilibrium at the timescale of larger than 100 fs (see Supplementary Fig. 16c).

Having obtained the XX^- formation rate, the Bohr radius a_{XX^-} can be calculated by the following equation

$$a_{XX^-} = \frac{\beta}{v_{XT}} \tag{S11}$$

where $v_{XT} = \sqrt{v_X^2 + v_T^2}$ is the relative velocity of the X and T, with 2D Maxwellian velocity of $v_X = \sqrt{\frac{\pi k_B T}{2m_X}}$ and $v_T = \sqrt{\frac{\pi k_B T}{2m_T}}$ respectively, $m_{X(T)} = 0.64\,(0.96) m_e$ is the X (T) mass, T= 4 K is the experimental temperature and k_B is the Boltzmann constant.

6.2 Transient absorption dynamics of X and XX^- in monolayer WS_2

To extract the XX^- formation rate, transient absorption spectrum of monolayer WS_2 is performed under 4K with a pump energy of 2.195 eV, as shown in Supplementary Fig. 16a. Since β may decrease at high pump fluence due to pump-induced heating effect, a relatively low pump fluence of 10 μJ cm^{-2} is used here. This fluence corresponds to a total injected exciton density of 3400 μm^{-2}, at which the nonlinear effect of all exciton complexes can be ignored (see Supplementary Fig. 15). Three well-separated photo-induced bleaching signals are found at the X, T and XX^- resonances in the first 1 ps (Supplementary Fig. 16b), which is attributed to their state filling effects. After 1 ps, however, a significant photo-induced absorption peak is found at the low-energy side of X and T. This feature peaks at the time delay of 4~5 ps followed by a slow decay with a lifetime of several hundred picoseconds, a typical timescale of lattice heating in WS_2[17]. Generally, lattice heating may result in a photo-induced absorption feature at low, red-shift energy, and simultaneously a photo-induced bleaching feature at high, original resonance. Thus, the X bleaching peak has two contributions with distinct timescales: phase space filling of exciton at the first 1 ps and lattice heating-induced band redshift after 1 ps. By integrating the signal over the whole photo-induced absorption and bleaching peaks (2.04 eV~2.10 eV), we can roughly eliminate the influence of the lattice heating, and acquire a pure state filling signal representing the X density, as shown in Supplementary Fig. 16c.

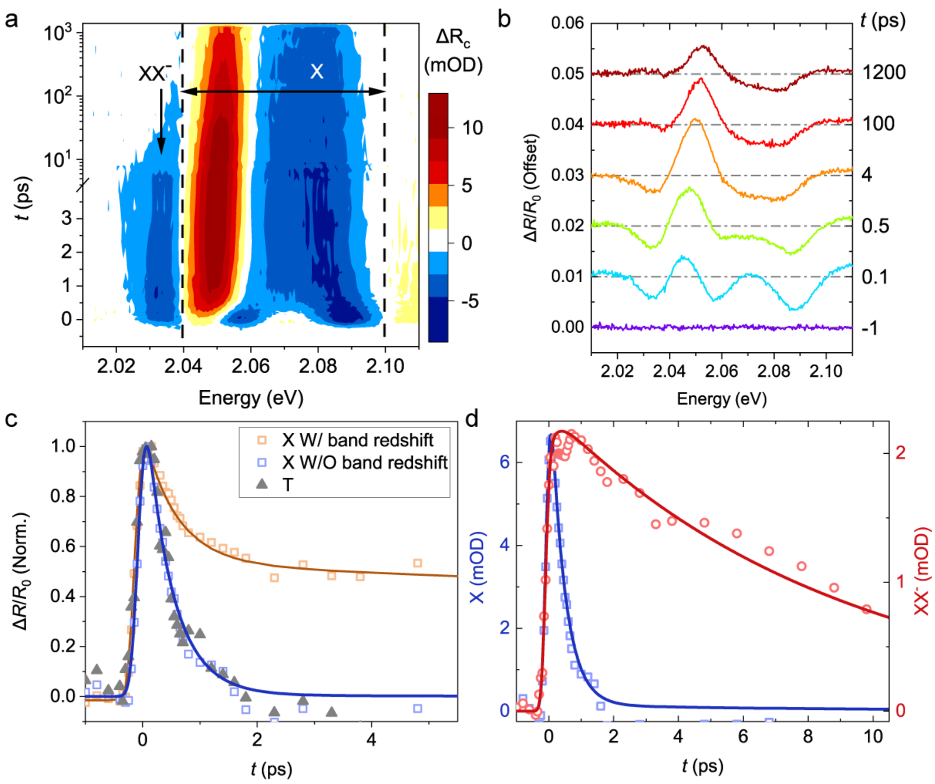

Supplementary Figure 16. Transient absorption spectrum of monolayer WS_2 measured at 4 K with a pump fluence of 10 μJ cm^{-2}. (a) 2D pseudo-color image, the blue region denotes the photo-induced bleaching, while the red region represents the photo-induced absorption. (b) Transient absorption spectrum at selected probe delays. (c) Normalized relaxation dynamics of the neutral exciton with (orange, the original X resonance peak) and without (blue, integrating from 2.04 eV to 2.10 eV, as marked in (a)) the band redshift component, and the trion dynamics (gray triangle, T resonance peak) is also provided for comparison. The bleaching signal at X resonance after 2 ps is almost completely caused by the lattice heating induced band redshift rather than the state filling of exciton. (d) Experimental (dots) transient absorption dynamics of X (considering the band redshift) and XX^- compared with the expected dynamics (lines) using the rate equation S10.

Having acquired the state filling induced X and XX^- dynamics, we extract the XX^- formation rate β from fitting the relaxation curves using equation S10, as shown in Supplementary Fig. 16d. Here, since the pump energy is far away from all the three exciton complexes, we assume that relative injecting density of the three exciton complexes is determined by their relative oscillator strength. The dynamics of both the X and XX^- are well reproduced by the rate equation, with the fitting parameter of $\beta = 0.8\ \mathrm{cm^2 s^{-1}}$, $\gamma_X^{-1} = 0.5\ \mathrm{ps}$, $\gamma_{XX^-}^{-1} = 9\ \mathrm{ps}$. The ultrafast relaxations of X and XX^- (and also T) are mainly attributed to nonradiative recombination through carrier trapping by the vast trap states at the surface of monolayer WS_2. The XX^- formation rate of $\beta = 0.8\ \mathrm{cm^2 s^{-1}}$ corresponds to a Bohr radius of $a_{XX^-} \approx 5\ \mathrm{nm}$, which is comparable to that estimated from PL emission dynamics using neutral biexciton configuration in WSe_2[16].

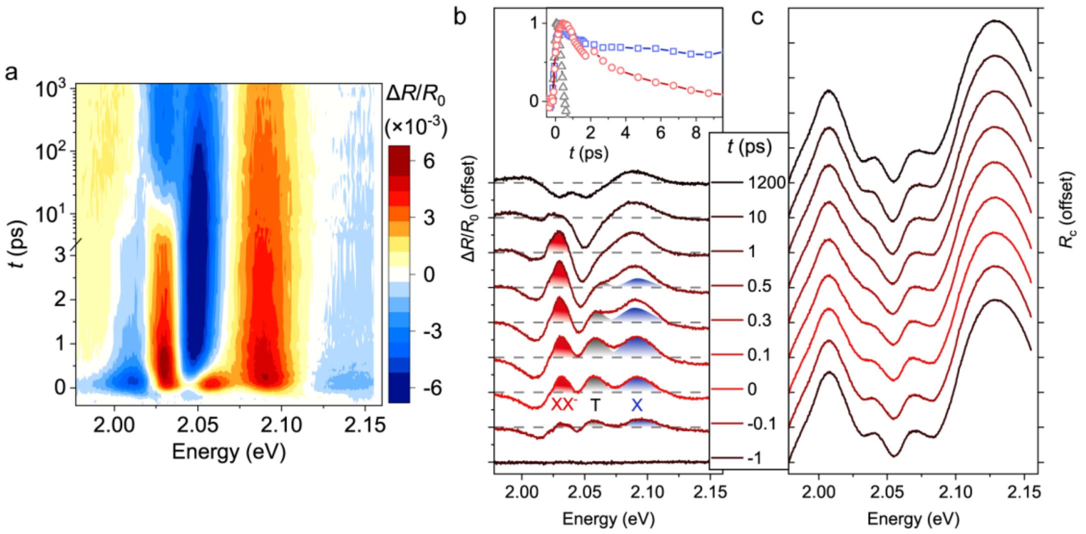

Supplementary Figure 17. Visualization of polariton nonlinearities and their dynamics at small injection density of ~3×10^4 μm^{-2}. (a) 2D pseudo-color image at pump energy of ~2.19 eV, the absorption features ($\Delta R > 0$) represent the pump-induced saturation nonlinearity. (b-c) $\Delta R/R_0$ spectrum (b) and transient R_c spectrum (c) at selected time delays. Inset of (b), time evolution of the normalized $\Delta R/R_0$ at the three excitonic resonances.

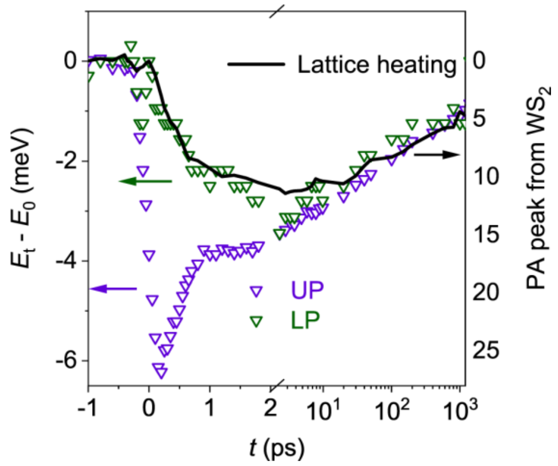

Supplementary Figure 18. Saturation nonlinearities and lattice heating induced band shifts of the UP and LP eigenstates. The data are extracted from Fig. 4d in the main text. The ultrafast red shift of UP (violet dots) before 1 ps is attributed to the saturation nonlinearity induced by coherent polariton (<100 fs) and incoherent exciton (100fs~1ps), while the slow red shifts of the UP (violet dots) after 1 ps and LP (green dots) in the whole time are caused by lattice heating, whose dynamics is depicted by the PA feature of

the transient absorption spectrum from pure WS_2 shown in Supplementary Fig. 16a (black line).

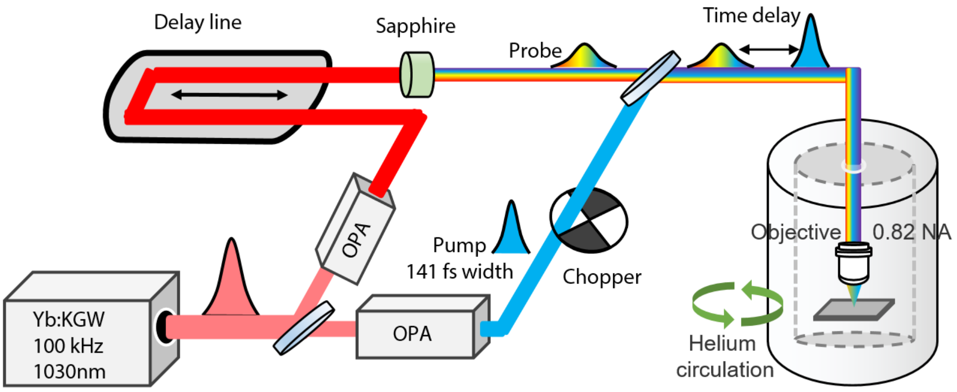

Supplementary Figure 19. sketch of the pump-probe experimental setup. OPA: optical parametric amplifier. NA: numerical aperture.

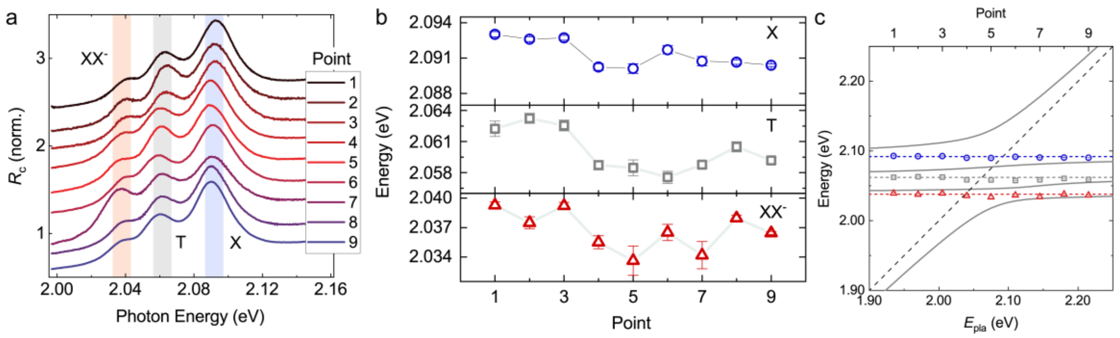

Supplementary Figure 20. Homogeneity of WS_2 sample. (a) R_c spectra of nine different WS_2 regions from the same batch of material. (b) Extracted resonant energy of X, T and XX^- from (a) by Lorentzian fitting. (c) Comparison between the polariton dispersions from Fig. 1d and the plotted excitonic energy from (b).

References:

1. Cuadra, J. et al. Observation of Tunable Charged Exciton Polaritons in Hybrid Monolayer WS_2−Plasmonic Nanoantenna System. *Nano Lett.* **18**, 1777-1785 (2018).
2. Zinkiewicz, M. et al. Excitonic Complexes in n-Doped WS_2 Monolayer. *Nano Lett.* **21**, 8943-8943 (2021).
3. Das, S. et al. Highly Nonlinear Biexcitonic Photocurrent from Ultrafast Interlayer Charge Transfer. *ACS Nano* **16**, 9728-9735 (2022).
4. Paur, M. et al. Electroluminescence from multi-particle exciton complexes in transition metal dichalcogenide semiconductors. *Nat. Commun.* **10**, 1709 (2019).
5. Barbone, M. et al. Charge-tuneable biexciton complexes in monolayer WSe_2. *Nat. Commun.* **9**, 3721 (2018).
6. Lundt, N. et al. The interplay between excitons and trions in a monolayer of $MoSe_2$. *Appl. Phys. Lett.* **112**, 031107 (2018).
7. Cadiz, F. et al. Ultra-low power threshold for laser induced changes in optical properties of 2D molybdenum dichalcogenides. *2D Mater.* **3**, 045008 (2016).

8. Fu, S. et al. Long-lived charge separation following pump-wavelength–dependent ultrafast charge transfer in graphene/WS_2 heterostructures. *Sci. Adv.* **7**, eabd9061 (2021).
9. Du, W. et al. Ultrafast Modulation of Exciton–Plasmon Coupling in a Monolayer WS_2–Ag Nanodisk Hybrid System. *ACS Photonics* **6**, 2832-2840 (2019).
10. Sang, Y. et al. Tuning of Two-Dimensional Plasmon–Exciton Coupling in Full Parameter Space: A Polaritonic Non-Hermitian System. *Nano Lett.* **21**, 2596-2602 (2021).
11. Tang, Y. et al. Interacting plexcitons for designed ultrafast optical nonlinearity in a monolayer semiconductor. *Light. Sci. Appl.* **11**, 94 (2022).
12. Zengin, G. et al. Realizing Strong Light-Matter Interactions between Single-Nanoparticle Plasmons and Molecular Excitons at Ambient Conditions. *Phys. Rev. Lett.* **114**, 157401 (2015).
13. Tang, Y. et al. Ultrafast Response of a Hybrid Device Based on Strongly Coupled Monolayer WS_2 and Photonic Crystals: The Effect of Photoinduced Coulombic Screening. *Laser Photonics Rev.* **14**, 1900419 (2020).
14. Cunningham, P. D., Hanbicki, A. T., McCreary, K. M. & Jonker, B. T. Photoinduced Bandgap Renormalization and Exciton Binding Energy Reduction in WS_2. *ACS Nano* **11**, 12601-12608 (2017).
15. Moody, G. et al. Intrinsic homogeneous linewidth and broadening mechanisms of excitons in monolayer transition metal dichalcogenides. *Nat. Commun.* **6**, 8315 (2015).
16. You, Y. et al. Observation of biexcitons in monolayer WSe_2. *Nat. Phys.* **11**, 477-481 (2015).
17. Ruppert, C., Chernikov, A., Hill, H. M., Rigosi, A. F. & Heinz, T. F. The Role of Electronic and Phononic Excitation in the Optical Response of Monolayer WS_2 after Ultrafast Excitation. *Nano Lett.* **17**, 644-651 (2017).
